# Evaluation of a Cost Effective In-House Method for HIV-1 Drug Resistance Genotyping Using Plasma Samples

**DOI:** 10.1371/journal.pone.0087441

**Published:** 2014-02-12

**Authors:** Devidas N. Chaturbhuj, Amit P. Nirmalkar, Ramesh S. Paranjape, Srikanth P. Tripathy

**Affiliations:** 1 Drug Resistance Lab, National AIDS Research Institute, Indian Council of Medical Research, Bhosari, Pune, India; 2 Department of Epidemiology & Biostatistics, National AIDS Research Institute, Indian Council of Medical Research, Bhosari, Pune, India; 3 National AIDS Research Institute, Indian Council of Medical Research, Bhosari, Pune, India; 4 National JALMA Institute Of Leprosy And Other Mycobacterial Diseases, Indian Council of Medical Research, Bhosari, Agra, India; McGill University AIDS Centre, Canada

## Abstract

**Objectives:**

Validation of a cost effective in-house method for HIV-1 drug resistance genotyping using plasma samples.

**Design:**

The validation includes the establishment of analytical performance characteristics such as accuracy, reproducibility, precision and sensitivity.

**Methods:**

The accuracy was assessed by comparing 26 paired Virological Quality Assessment (VQA) proficiency testing panel sequences generated by in-house and ViroSeq Genotyping System 2.0 (Celera Diagnostics, US) as a gold standard. The reproducibility and precision were carried out on five samples with five replicates representing multiple HIV-1 subtypes (A, B, C) and resistance patterns. The amplification sensitivity was evaluated on HIV-1 positive plasma samples (n = 88) with known viral loads ranges from 1000–1.8 million RNA copies/ml.

**Results:**

Comparison of the nucleotide sequences generated by ViroSeq and in-house method showed 99.41±0.46 and 99.68±0.35% mean nucleotide and amino acid identity respectively. Out of 135 Stanford HIVdb listed HIV-1 drug resistance mutations, partial discordance was observed at 15 positions and complete discordance was absent. The reproducibility and precision study showed high nucleotide sequence identities i.e. 99.88±0.10 and 99.82±0.20 respectively. The in-house method showed 100% analytical sensitivity on the samples with HIV-1 viral load >1000 RNA copies/ml. The cost of running the in-house method is only 50% of that for ViroSeq method (112$ vs 300$), thus making it cost effective.

**Conclusions:**

The validated cost effective in-house method may be used to collect surveillance data on the emergence and transmission of HIV-1 drug resistance in resource limited countries. Moreover, the wide applications of a cost effective and validated in-house method for HIV-1 drug resistance testing will facilitate the decision making for the appropriate management of HIV infected patients.

## Introduction

Close to 60% of those who require anti-retroviral treatment (ART) are already under ART [1]. The long term success of this programme will depend on the continued virus suppression as a result of anti-retroviral treatment. However, in resource-limited countries, HIV drug resistance testing is not generally available or it is too costly to be used in the routine monitoring of patients receiving ART. Monitoring the trends of HIV drug resistance among treatment failures is crucial as HIV drug resistance is a major threat with ability to negate the benefits accrued by the free ART programme. Detection and monitoring of HIV drug resistance by molecular genotyping is pivotal to ensure ongoing regimen efficacy. Therefore, the World Health Organization (WHO) recommends population-based surveillance and monitoring of HIV drug resistance in resource-limited settings [2], [3]. The reported pattern and rates of transmitted and acquired drug-resistant HIV variants will collectively form the regional and global recommendations on which ART is to be maintained or changed in the first and second-line antiretroviral regimens [4]. The ViroSeq Genotyping System 2.0 (Celera Diagnostics, US) and TruGene are the two FDA-approved commercially available methods for HIV drug resistance testing [5], [6]. The majority of the HIV-1 infected patients cannot afford the commercial HIV-1 drug resistance testing when they experience ART failure because of its high cost. Thus, there is a need for the development of a cost effective and efficient in-house method for HIV-1 drug resistance testing for application in resource limited settings.

The development of an in-house method has been done independently in each lab, usually requiring investment of considerable effort to optimize the procedures used [7]–[11]. In larger studies and surveys, it is important to have confidence that the results generated from different participating labs are of high quality and comparable to each other. One solution to this problem would be to recommend all labs to use the same method, but this is not practical, given that the local difference in reagent supply, HIV subtypes, personnel training and the requirement to change the established procedure may vary. An alternative approach is to use a validated method that will ensure quality results.

The validation of an in-house method is now a prerequisite for testing any sample for World Health Organization (WHO) recommended surveillance and studies [12]. Most of the laboratories have validated the in-house method by the minimal criteria strategy which includes the comparison of the sequence of large numbers of specimens (e.g. 50 to 200) obtained by an in-house method with that obtained by previously validated commercially available FDA approved HIV genotyping method as “Gold Standard” [7], [9], [10]. Whereas this minimal validation procedure can describe the accuracy of the method, further assessment of the other analytical characteristics such as reproducibility, precision and analytical sensitivity is also required to ensure the reliable results. The acceptability of the performance of a method is determined by the analytical performance characteristics such as accuracy, reproducibility, precision, amplification sensitivity and specificity.

In this study, previous in-house method [13] was modified and evaluated by the analytical performance characteristics such as accuracy, reproducibility, precision, analytical sensitivity.

## Materials and Methods

### Samples

The validation of an in-house method was done according to the WHO guidelines [12] including participation in Virological Quality Assessment (VQA) HIV Genotypic Drug Resistance proficiency testing panels (VQA contract # NO1-AI-50044). The accuracy of the in-house method was assessed by comparing 26 paired VQA HIV Genotypic Drug Resistance proficiency testing panel sequences. The nucleotide/amino acid sequence obtained by the ViroSeq method (Celera Diagnostics, US) was considered as the gold standard with which the nucleotide/amino acid sequences obtained by the in-house method were compared.

The samples used in the validation study had similar characteristics to the samples which are tested routinely (sample type: plasma, genetic subtype: HIV-1 subtype C, A and B, viral load range >1000 copies/ml, resistance pattern: reverse transcriptase and protease inhibitor mutations). The reproducibility and the precision were carried out on five samples with five replicates each. All these samples were previously tested for HIV-1 drug resistance genotyping using the ViroSeq method and aliquots were stored at −70±5°C.

### Validation criteria

Accuracy was defined as detection of 99% of known HIV-1drug resistance mutations when compared with the results obtained by the ViroSeq Genotyping System 2.0 (Celera Diagnostics, US). The reproducibility and precision were defined as ≥98% nucleotide identities in ≥90% of pairwise comparisons, with the mixtures being counted as partially discordant. The nucleotide/amino acid sequences were analyzed under three categories: [10] (i) Concordant (if both the ViroSeq and in-house method gave the same nucleotide/amino acid). (ii) Partially discordant (if nucleotide base/amino acid mixture by one method but not by the other) and (iii) Discordant (if the two methods detected different nucleotide bases/amino acids).

### Analytical Sensitivity

Since HIV-1 subtype C is the predominant subtype in India, the analytical sensitivity of the in-house method was tested by using two HIV-1 subtype C clinical samples with the viral load 75,520 copies/ml and 18,500 copies/ml determined by the COBAS Amplicor HIV-1 Monitoring Kit version 1.5 (Roche Diagnostics, Branchburg, New Jersey). The samples were serially diluted (the created copy number ranged from 4720 to less than 1000 HIV-1 RNA copies/ml) using HIV negative human plasma. Viral RNA was then extracted and the *pol* gene was amplified by the in-house method as described below.

The protocol was finalized before initiation of the evaluation of the in-house HIV drug resistance assay. As per the WHO guidelines, an acceptance criterion for each analytical performance characteristic was established in advance [12]. The protocol for the in-house method used for the validation is described below.

### RNA Extraction

RNA extraction from plasma samples were performed using the NucliSENS® easyMAG™ (Biomerieux, Durham, NC) automated nucleic acid extraction system according to the manufacturer's recommendations (NucliSENS easyMAG user manual, v 1.1; BioMérieux, Boxtel, Netherlands). Five hundred microlitres of each sample was placed in the disposable sample vessel and the sample vessel was loaded onto the extractor. After the initial lysis incubation for 10 min, 50 µl of magnetic silica was added to each sample and the extractor was restarted. The samples were eluted in 50 µl of extraction buffer 3. All samples were transferred to a 1.5-ml microcentrifuge tube and stored at −70°C.

### RT-PCR and Nested PCR

RT-PCR was performed as described previously [13]. The nested PCR was performed using the inner primer pair as mentioned in Table 1 to get an amplified fragment of 1204 bp. The amplification was done using Taq DNA polymerase (Genei, Bangalore India) 3 U/µl (1 µl) in a 10× PCR buffer B (5 µl), 25 mM MgCl2 (4 µl), 2 mM dNTPs mix (Fermentas) (5 µl), 10 pmol of each primer and 6 µl of RT-PCR product. The final volume of 50 µl reaction was made up using DNase/RNase free water. The reaction conditions used for nested PCR were: initial denaturation at 94°C for 2 min followed by DNA amplification: 25 cycles at 94°C for 20 sec, 59°C for 45 sec, 72°C for 3 min and a final extension for 10 min at 72°C.

**Table 1:** Primers used in the In-house method for HIV-1 drug resistance genotyping.

Primers name	Sequence (5′>3′)	HXB2 Position
RT-PCR		
2021F	AAGGCTGTTGGAAATGTGG	2021>2039
4521R	RCTGTTTCTTGTCCTGTTTCTGC	4522<4500
Nested PCR		
2135F	TTTAGAGCAGACCAGAGCCAACAGC	2135>2159
3338R	TTTTCCCACTAACTTCTGTATAGTCATTG	3311<3338
Sequencing		
2135F	TTTAGAGCAGACCAGAGCCAACAGC	2135>2159
2493F	CCTGTCAACATAATTGGAAG	2493>2512
3012F	GGATCACCAGCAATATTC	3012>3029
2557R	GGTACAGTTTCAATGGGAC	2557<2575
3117R	CCCTATTTCTAAGTCAGATCC	3117<3137
3338R	TTTTCCCACTAACTTCTGTATAGTCATTG	3311<3338

A GeneAmp PCR system 9700 thermal cycler (Applied Biosystem, CA, USA) was used for all PCRs. The results were checked by electrophoresis of the nested PCR products on 1% Agarose (GeNei, Bangalore, India) gels containing ethidium bromide (Sigma Aldrech, USA) and visualization of the amplified bands under UV light.

The amplified PCR products were purified using the QIAquick PCR purification kit (Qiagen Hilden, Germany) and eluted in 30 µl elution buffer. The purified PCR product was directly sequenced in both directions using BigDye Terminator Cycle Sequencing Ready Reaction kit version 3.1 (Applied Biosystem, CA, USA).

### Sequence analysis

DNA sequencing was performed on 3100 DNA genetic analyzer (Applied Biosystem, CA, USA) with the six specific primers mentioned in Table 1. These sequencing primers provided overlapping, bidirectional sequences covering the whole protease region and partial reverse transcriptase region. The sequencing reaction was performed in 10 µl volume containing 2 µl ready reaction mix, 4 pmol primers, 2 µl of 5× sequencing buffer, 2 µl of purified PCR product (30 ng/µl) and the volume was adjusted to 10 µl with DNase/RNase free water. The sequencing reaction was carried out using 25 linear amplification cycles of 96°C for 10 sec, 50°C for 5 sec, 60°C for 4 min. Unincorporated dNTPs were then removed by precipitation with 80% isopropanol and the dried pellet was resuspended in 10 µl of Hi-DiTM formamide (Applied Biosystem, CA, USA). The raw sequence data from ABI 3100 genetic analyzer was assembled, aligned and edited with the SeqScape v2.0 software (Applied Biosystem, CA, USA).

To rule out PCR contamination, the phylogenetic tree was generated by using the PhyML software to create a maximum likelihood tree [14]. The reference sequences were obtained from the Los Alamos HIV Database (www.hiv.lanl.gov). The sequence quality was also checked by Sequence Quality Assessment Tool (SQUAT) analysis software [15]. The final edited sequences were submitted to the “HIVdb Program: Sequence Analysis” in the Stanford University HIV drug resistance database for drug resistance interpretation [16]. Pairwise nucleotide sequence identity and discrepancy were analyzed using BioEdit v 7.0.9.0 [17]. The Rega HIV-1 subtyping tool Version 2.0 was used to determine the HIV-1 subtype (http://dbpartners.stanford.edu/RegaSubtyping/ Accessed on 14^th^ May 2012).

### Statistical analysis

Wilcoxon Signed-Rank test was used to analyze the difference in number of nucleotide mixtures detected between the ViroSeq and in-house method. The statistical significance was considered when P value was <0.05.

## Results

### Accuracy

All 26 paired VQA HIV Genotypic Drug Resistance proficiency testing panel samples were amplified and sequenced successfully using both the in-house and ViroSeq methods. The mean nucleotide identity was 99.41±0.46% (mean ± SD) among paired nucleotide sequences (Table 2). Wilcoxon signed-rank test was used to compare the in-house and ViroSeq method basecalling for mixed bases and revealed no significant difference (P, 0.382).

**Table 2:** Pairwise sequence identity analysis between the in-house and the ViroSeq method.

VQA panel	In-house vs ViroSeq
# of samples	26
% Nucleotide identity	99.41±0.46
Mean nucleotide mixture	10.12 vs 10.00
% amino acid identity	99.68±0.35
# of DR mutations	135 vs 120
Partial discordant mutations (%)	15 (11.11)

A total of 135 drug resistance-associated amino acid positions in protease (36 mutations) and reverse transcriptase (99 mutations) region were detected among 26 paired sequences. There was no complete nucleotide or amino acid discordance. The overall amino acid codon agreement was 99.68±0.35% among paired amino acid sequences.

The partial discordance due to synonymous/non-synonymous substitutions was observed at 15 amino acid positions including 2 positions in protease and 13 positions in the reverse transcriptase region (Table 3). Out of 15 partial discordant positions, in 13 positions mixtures of the wild type and the mutant virus codons were detected by the in-house method only, while the ViroSeq method detected either the wild type or mutated codon. In the remaining two positions, mixtures of the wild type and the mutant virus codons were detected by the ViroSeq method only, while the in-house method could detect only the wild type or mutated codon.

**Table 3:** Drug resistance-associated amino acid positions in protease and reverse transcriptase from 26 proficiency testing panel plasma samples genotyped by the in-house and the ViroSeq methods.

Amino Acid	Amino acid detected by the in-house method (# of samples)	Amino acid detected by the ViroSeq method (# of samples)	# of partially discordant mutations
Protease			
**L10**	**L (21), I (4), IL (1)**	**L (21), I (5)**	**1**
L23	L (23), I (3)	L (23), I (3)	0
L33	L (23), F (3)	L (23), F (3)	0
M46	M (23), L (3)	M (23), L (3)	0
**I47**	**I (25), IV (1)**	**I (26)**	**1**
I54	I (23), V (3)	I (23), V(3)	0
A71	A (21), V (2), T (1), TI (2)	A (21), V (2), T (1), TI (2)	0
G73	G (24), GS (2)	G (24), GS (2)	0
V82	V (23), A (3)	V (23), A (3)	0
N88	N (23), G (3)	N (23), G (3)	0
L90	L (21), M (5)	L (21), M (5)	0
Reverse Transcriptase			
M41	M (19), L (7)	M (19), L (7)	0
A62	A (21), V (3), AV (2)	A (21), V (3), AV (2)	0
K65	K (24), KR (2)	K (24), KR (2)	0
D67	D (18), N (5), DN (2)	D (17), N (5), DN (2)	0
T69	T (24), IT (2)	T (24), IT (2)	0
K70	K (24), R (2)	K (24), R (2)	0
**L74**	**L (21), V (2), I (1), IL (2)**	**L (23), V (1), I (1), IV (1)**	**2**
**V75**	**V (22), T (2), AV (1), AITV (1)**	**V (22), T (2), AITV (2)**	**1**
**V90**	**V (23), I (2), IV (1)**	**V (23), I (2), IV (2)**	**1**
L100	L (23), I (3)	L (23), I (3)	0
**K101**	**K (23), Q (2), KPQT (1)**	**K (24), Q (2)**	**1**
**K103**	**K (11), N (13), KR (2)**	**K (13), N (13)**	**2**
V106	V (22), M (4)	V (22), M (4)	0
E138	E (24), A (2)	E (24), A (2)	0
V179	V (24), D (2)	V (24), D (2)	0
**M184**	**M (10), V (8), L (3), MV (5)**	**M (14), V (8), L (3), MV(1)**	**4**
**Y188**	**Y (24), C (1), CY (1)**	**Y (24), C (2)**	**1**
L210	L (23), W (3)	L (23), W (3)	0
**T215**	**T (19), Y (5), CS(1), ST (1)**	**T (20), Y (5), C (1)**	**1**
H221	H (23), Y (3)	H (23), Y (3)	0
P225	P (24), H (1), HP (1)	P (24), H (1), HP (1)	0

Note: Partial discordant positions are shown in bold.

### Reproducibility

**Figure 1:**
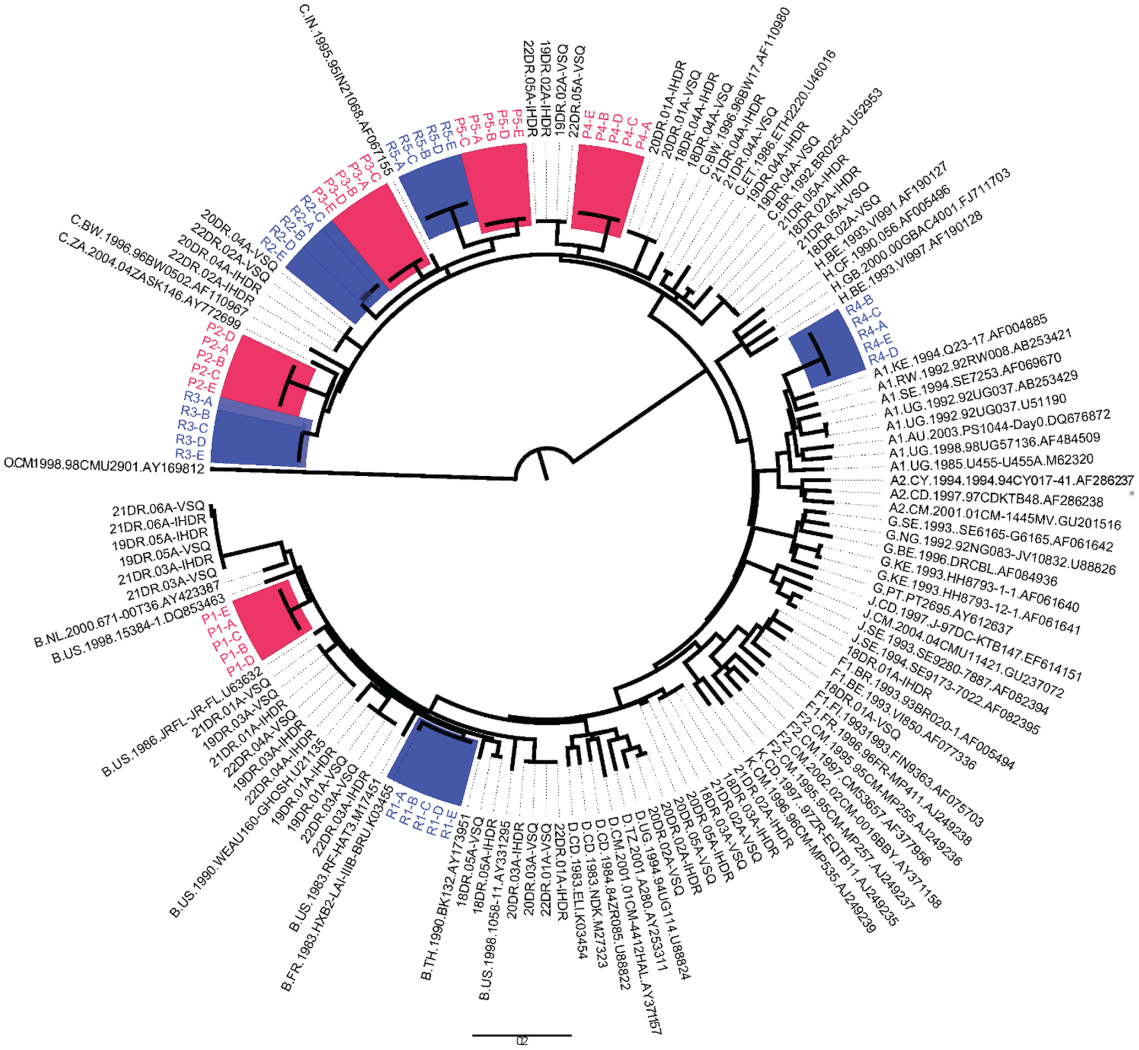
Phylogenetic analysis of the sequences generated during the evaluation of an in-house method. The phylogenetic tree was generated by using the PhyML software to create a maximum likelihood tree [13]. The reference sequences were obtained from the Los Alamos HIV Database (www.hiv.lanl.gov). IHDR- sequences generated by an in-house method; VSQ- sequences generated by the ViroSeq method; R1 to R5- sample used in the reproducibility study (hilighted in blue); P1 to P5- sample used in the precision study (hilighted in red); A, B, C, D, E are the replicates of the same sample.

All five replicates of five samples were amplified and sequenced successfully. The drug resistance mutation pattern and subtype distribution of samples selected for reproducibility study are given in Table 4. A total of 25 sequences were generated from five samples. The phylogenetic tree for all 25 sequences along with HIV-1 reference sequences (n = 56) was constructed by using the PhyML software to create a maximum likelihood tree [14]. The sequences confirmed the absence of cross-contamination or sample mix-up. The paired sequences obtained for each sample were more closely related to one another than to the sequences of any other sample (Fig. 1). Moreover, as expected, sequences from the same sample clustered together.

**Table 4:** Reproducibility and Precision of an in-house method.

				Replicate Tests						
Sample ID	HIV-1 VL (Copies/ml)	HIV-1 Subtype	% Nucleotide sequence identity	No. of drug resistance mutations					# of Partially discordant mutations	# of Discordant mutations
				A	B	C	D	E		
Reproducibility										
R1	1,00,000	B	100.00±0.00%	0	0	0	0	0	0	0
R2	12,715	C	99.72±0.20%	2	2	2	2	2	0	0
R3	2,808	C	99.88±0.05%	3	3	3	3	3	0	0
R4	6,65,000	A	99.87±0.10%	12	12	12	12	12	0	0
R5	97,189	C	99.92±0.05%	18	18	18	18	18	0	0
Precision										
P1	76,600	B	99.97±0.05%	2	2	2	2	2	0	0
P2	Not Available	C	100.00±0.00%	14	14	14	14	14	0	0
P3	14,393	C	99.49±0.40%	3	3	3	3	3	0	0
P4	6,29,757	C	99.87±0.11%	8	8	8	8	8	0	0
P5	Not Available	C	99.77±0.18%	10	10	10	10	10	0	0

We found high nucleotide sequence identities ranging from 99.72% to 100.00% (99.88±0.10) (Table 4). The minor differences observed in sequence identity were caused by base mixtures (nucleotide mixture by one method but not by another). However, this difference did not translate into differences in amino acids.

A total of 35 drug resistance-related positions [PI mutations (7), NRTI mutations (16) and NNRTI mutations (12)] for each sequence and its replicates were also analyzed for reproducibility. All 35 drug resistance mutations were found to be reproducible with 100% concordance.

### Precision

The precision study also showed high nucleotide sequence identity ranging from 98.49% to 100.00% (99.82±0.20) (Table 4). A total of 34 drug resistance-related positions [PI mutations (16), NRTI mutations (12) and NNRTI mutations (6)] were seen for five replicates of five samples analyzed for precision study. In addition, one sample showed an insertion at amino acid 35 in the protease for all replicates. All 34 drug resistance mutations and one insertion at amino acid 35 in the protease were found to be reproducible with 100% concordance. The drug resistance mutation pattern and subtype distributions of samples selected for precision study is given in Table 4.

### Amplification Sensitivity

The amplification sensitivity of the in-house method was evaluated on HIV-1 positive plasma samples (n = 88) with known viral load ranging from 1000 to 1.8 million RNA copies/ml (see Table S1) for the viral copy number range, % amplification and subtype distribution). All the samples with viral load >1000 HIV-1 RNA copies/ml were amplified and sequenced successfully.

We have also evaluated the amplification sensitivity by serial dilution of two samples with high viral loads to achieve a range of copy numbers (4720, 2360, 1180, 590 RNA copies/ml) using plasma from HIV-1 negative donor followed by four replicate testings of each dilution. The amplification was seen in all four replicates of the sample with >1000 RNA copies/ml, whereas amplification was observed only in two out of four replicates with <1000 RNA copies/ml for both samples.

## Discussion

In this study, the validation of an in-house method for HIV-1 drug resistance genotyping developed at the National AIDS Research Institute (ICMR), Pune, India was orchestrated. Accuracy of the in-house method was assessed by comparing the results of VQA HIV Genotypic Drug Resistance proficiency testing panel samples generated by the in-house method with the results of the ViroSeq method. The VQA HIV Genotypic Drug Resistance proficiency testing panel samples used in this study consisted of HIV-1 group M subtypes with known viral loads (viral load ranges 3000 to 60,633 copies/ml). All these samples (n = 26) were amplified and sequenced successfully by both in-house method and ViroSeq method with high nucleotide sequence identity (99.41±0.46%). All the clinically relevant mutations were concordant by both methods and reproducible. Despite the minimal differences seen by the partial discordance in nucleotide base callings, the in-house method demonstrated an ability to identify clinically relevant mutations correctly (99.41±0.46) when compared with the ViroSeq method.

In every case of nucleotide partial discordance, one method detected a mixture of the wild type and the mutant virus, the other method detected either the wild type or the mutant virus. We performed sequence editing for all validation samples using the minor peak default nucleotide mixture calling setting at 30% of the major peak in bidirectional sequences. The minor peaks at partial discordant nucleotide could be seen, but were below the nucleotide mixture cutoff (30%) on the chromatogram by SeqScap software and ViroSeq Software. Thus, these mixtures were not counted, resulted in the partial discordance. These results indicated that the sequences generated by population-based sequencing were highly reproducible but the sensitivity at detecting low viral variants was very challenging. Many factors could contribute to the detection of mixed nucleotide sites including viral quasispecies, primer binding preference and location, Taq polymerase misincorporation and sequence quality. The sequence identity and codon concordance are challenging when mixed nucleotide bases are present [18]. It has been reported that the ViroSeq method detected more mixtures of the wild type with the mutant virus (78%) than seen in the in-house assay (22%) [10]. In contrast, our results revealed no significant difference (P value, 0.382) in basecalling for nucleotide mixed bases. The variability in detecting nucleotide mixtures of the wild type or the mutant virus was likely due to the first-round RT-PCR in sampling of quasispecies strains rather than technical errors in the sequencing process [19], [20].

Further, the in-house method was assessed for inter and intra-assay variability (reproducibility and precision) on multiple aliquots of the same sample in different test runs or within a test run respectively. The samples selected for inter and intra-assay variability were from multiple HIV subtypes (C, A and B) and contained the most common NRTI mutations (M184V & D67N), NNRTI mutations (K103N, G190A) and PR mutations (M46I, I54V & L90M). The inter-assay variability was analyzed over time (24 weeks), among different lots of critical reagents (Extration buffers, RT PCR kit and Magnetic Silica). The high degree of pairwise nucleotide sequence identity for inter and intra-assay comparisions (99.88±0.10 and 99.82±0.20 respectively) showed a high degree of reproducibility of the result.

The laboratory has already been certified for HIV Genotypic Drug Resistance using the ViroSeq method and is under VQA HIV Genotypic Drug Resistance proficiency testing program. However, the validated in-house method also successfully completed a total of five VQA HIV Genotypic Drug Resistance proficiency panels, resulting in the in-house method described here being certified by the Virology Quality Assessment (VQA) Program funded by National Institute of Health (NIH). The successful testing of VQA HIV Genotypic Drug Resistance proficiency panels indicated that the in-house method could genotype HIV-1 group M subtypes.

The manpower and turnaround time for both the ViroSeq and the in-house methods were similar. The cost per test incurred for the in-house method (US 112$) was approximately 50% of the cost incurred using the ViroSeq method (US 300$).

In conclusion, we have evaluated a cost effective in-house method for HIV-1 drug resistance testing using plasma samples. The validated in-house method was broadly sensitive in genotyping multiple HIV-1 group M subtypes. The validation analyses indicate 100% amplification sensitivity for samples >1000 HIV-1 copies/ml, and high accuracy (99.41±0.46) when compared with the ViroSeq method. In the present study, high degree of reproducibility (99.88±0.10) and precision (99.82±0.20) were also observed with the in-house method. The validated in-house method may be used to effectively monitor patients failing ARV therapy, as well as to collect surveillance data on the emergence and transmission of HIV-1 drug resistance isolates in resource limited countries. Moreover, the wide applications of a cost effective and validated in-house method for HIV-1 drug resistance testing will facilitate the decision making for the appropriate management of HIV infected patients and thereby reduce the risk of onset of further drug resistance-related mutations.

## Supporting Information

Table S1 **Supporting Information for the amplification sensitivity of an in-house method using known viral copy number samples.**
(DOCX)
