# Supplementary material for: Evaluation of a Cost Effective In-House Method for HIV-1 Drug Resistance Genotyping Using Plasma Samples
Source: PLoS One. 2014 Feb 12;9(2):e87441. doi: 10.1371/journal.pone.0087441 (PMC3922725; doi:10.1371/journal.pone.0087441)
Supplement: Table S1 — Supporting Information for the amplification sensitivity of an in-house method using known viral copy number samples. (DOCX) [file pone.0087441.s001.docx]

**SUPPLEMENTARY INFORMATION**

Table S1 : Amplification sensitivity using known viral copy number samples (n=88)

| **Viral load (copies/ml)** | **Number of samples (n)** | **Viral copy number Median** | **First Quartile** | **Third Quartile** | **Interquartile Range** | **HIV-1 Subtype distribution** | **Amplification% (n)** | **No Amplification% (n)** |
| --- | --- | --- | --- | --- | --- | --- | --- | --- |
| 1000 to 2,000 | 8 | 1499 | 1344.5 | 1499.5 | 293 | C (7) | 100 (8) | 0 (0) |
| 2001 to 5,000 | 7 | 3000 | 2135 | 4194 | 2059 | C (5), B (2) | 100 (7) | 0 (0) |
| 5,001 to 10,000 | 17 | 8020 | 5836.5 | 9056.5 | 3220 | C (4), B (11), A (1), D (1) | 100 (17) | 0 (0) |
| 10,001 to 50,000 | 31 | 19356 | 14393 | 37910 | 23517 | C (28), B (2), F (1) | 100 (31) | 0 (0) |
| 50,001 to 100, 000 | 9 | 75520 | 55757.5 | 88825.5 | 33068 | C (8), A (1) | 100 (9) | 0 (0) |
| Greater than 100,000 | 16 | 230000 | 148389.5 | 386062 | 237672.5 | C (15) , A (1) | 100 (16) | 0 (0) |
| Total | 88 | 16746.5 | 7548.5 | 58466.5 | 50198 | C (67), B (15), A (3), D (1), F (1) | 100 (88) | 0 (0) |
